# Pregnant couples’ attitude toward extended pre-conceptional genomic screening

**DOI:** 10.3325/cmj.2024.65.189

**Published:** 2024-06

**Authors:** Mojca Čižek Sajko, Bernarda Prosenc, Lovro Vidmar, Gordana Njenjić, Paula Duff, Borut Peterlin

**Affiliations:** 1 Clinical Institute for Genomic Medicine, University Medical Center Ljubljana, Ljubljana, Slovenia; 2 Clinical Department of Perinatology, Division of Gynecology and Obstetrics, University Medical Center Ljubljana, Ljubljana, Slovenia; 3 Faculty of Health Sciences, University of Novo Mesto, Novo Mesto, Slovenia; * The authors contributed equally.; Čižek Sajko et al: Pregnant couples’ attitude towards extended pre-conceptional genomic screening

## Abstract

**Aim:**

To determine the attitudes of pregnant couples toward carrier screening genomic tests.

**Methods:**

A validated 22-item questionnaire was offered in person by medical staff to pregnant women ≥32 weeks’ gestation and their partners attending prenatal classes from May to July 2014. The questionnaire inquired about demographic data, interest in various forms of genetic carrier screening tests, and genetic literacy.

**Results:**

Of 497 respondents, 69% expressed strong interest in carrier screening. The interested respondents exhibited substantial support for screening for common (82%) or all known genetic diseases (79%), as well as for treatable (79%) and untreatable diseases (85%). The majority of respondents believed that genetic test results could provide them with a sense of security but also provoke anxiety and fear. They were aware that these results could affect their perspective on life, work, and the atmosphere within their family, and acknowledged the potential effect on their relationship with their partner. However, none of these concerns diminished their desire to learn about their carrier status. Respondents with higher genetic literacy exhibited greater interest in screening tests (*P* = 0.006). More non-religious respondents compared with practicing religious respondents (*P* = 0.002), and more respondents with higher education compared with those with lower education, expressed interest in screening (*P* = 0.003).

**Conclusion:**

Most respondents expressed considerable interest in receiving information about their carrier status through genetic tests.

Carrier screening is a genetic testing method used to identify individuals, typically asymptomatic ones, who carry a gene mutation for an autosomal recessive or X-linked recessive disorder. Historically, carrier screening focused on the most prevalent recessive disorders, such as cystic fibrosis, Tay-Sachs syndrome, and thalassemia (1-3). Advancements in genotyping technology and reduced costs have expanded the range of disorders that can be screened for. Today, many commercial laboratories worldwide offer carrier screening panels that include over 100 less common or even extremely rare disorders (4,5). The expanded carrier screening approach has sparked discussions on technical, ethical, legal, and social considerations (6,7). Carrier screening, whether offered by commercial entities or national health care systems, should only be provided to target populations following responsible implementation procedures (8-10). This includes conducting thorough research on public attitudes and preferences.

The primary goal of carrier screening is to facilitate informed reproductive decision-making (8). Consumers should receive comprehensive information on the pros and cons of extended carrier screening. Pre-conceptional carrier screening, akin to genetic testing in general, has garnered substantial public support (8,11-13). Nevertheless, there have been instances where individuals do not express a desire for carrier screening (14). Previous studies have predominantly focused on more common and severe disorders within populations where recessive diseases are prevalent. The focus of the present study is to determine the pregnant couples’ attitude toward extended carrier screening, with a specific emphasis on discerning the factors associated with participation in pre-conceptional carrier couple screening.

## RESPONDENTS AND METHODS

### Study design and respondents

We developed a questionnaire and distributed it anonymously among pregnant women of Slovene nationality and their partners attending prenatal classes at the Gynecological Clinic in Ljubljana from May to July 2014. Prenatal classes offer education for women in the later stages of pregnancy, covering topics such as childbirth, newborn care, and relevant information. Pregnant women receive oral invitations to attend these classes during their regular check-ups with their gynecologist. The questionnaires were distributed personally to the attendees during one of the sessions, and partners did not need to be present. Respondents completed the questionnaire simultaneously in a classroom setting, with qualified personnel available to clarify any questions if necessary. Respondents were encouraged to thoughtfully consider the questions, and most of them completed the questionnaire in about 30 minutes. All participants in the prenatal classes willingly agreed to participate in the survey. The study was approved by the Medical Ethics Committee of the Republic of Slovenia (86/03/14).

### Questionnaire

The questionnaire was developed by a multidisciplinary research group comprising four clinical geneticists, two nurses, and a psychologist. The validity and reliability of the research instrument were tested through quantitative analysis. Construct validity assessed by exploratory factor analysis revealed five factors/concepts, with acceptable to good internal consistency (Cronbach’s alpha from 0.62 to 0.87).

The questionnaire consisted of three parts. The first part inquired about demographic data (Table 1). The second part consisted of 15 statements designed to assess participants' general knowledge about genetic disorders. The first statement addressed the general prevalence of genetic/heritable diseases, with participants receiving zero points for selecting “very rare (less than 1/1,000,000),” one point for “rare (less than 1/10,000),” and two points for “frequent (more than 1/1000)”. Subsequently, a series of statements assessed participants' perceptions of the heritability of 14 disease groups (eg, cancer, epilepsy, infections, deafness, infertility, hemophilia, neuro-muscular diseases, etc). The responses ranged from “1 - the disease is not at all heritable” to “5 - the disease is highly heritable.” Participants were awarded a point if their answer deviated by no more than one point from the norm, which was established as the mode of answers provided by five medical doctors working as genetic counselors. The total score for genetic literacy ranged from 0 to 16 points.

**Table 1:** The respondents' characteristics

	No. (%) of respondents		
Characteristic	female (n = 299)	male (n = 198)	total (n = 497)
Age, years			
18-25	33 (11)	12 (6)	45 (9)
26-35	212 (71)	137 (69)	349 (70)
36-45	54 (18)	47 (24)	101 (20)
>45	0 (0)	2 (1)	2 (0.4)
Education			
elementary school	3/298 (1)	3/195 (2)	6/493 (1)
high school	87/298 (29)	85/195 (44)	172/493 (35)
college or university	208/298 (70)	107/195 (55)	315/493 (64)
Pregnancies			
first	231 (77)	155/187 (83)	386/486 (79)
second	41 (14)	18/187 (10)	59/486 (12)
third or more	27 (9)	14/187 (7)	41/486 (8)
Genetic diseases in family history			
yes	44/298 (15)	21/194 (11)	65/492 (13)
no	254/298 (85)	173/194 (89)	427/492 (87)
Religiosity			
yes, practicing	72/278 (26)	46/182 (25)	118/460 (26)
yes, non-practicing	91/278 (33)	49/182 (27)	140/460 (30)
no	115/278 (41)	87/182 (48)	202/460 (44)
Genetic literacy score (points)			
0-4	11 (4)	3 (2)	14 (3)
5-8	64 (22)	45 (23)	109 (22)
9-12	138 (47)	93 (48)	231 (47)
13-16	80 (27)	53 (27)	133 (27)

The third part constituted the main section of the questionnaire and consisted of one closed question and 21 Likert-type statements. Respondents were asked to rate their level of agreement from 1 (“I totally disagree/I am not at all interested”) to 5 (“I totally agree/I am very interested”). In conjunction with question 22, the respondents were presented with the following informative paragraph: “Pre-conception genetic screening is performed on healthy parents-to-be. With it, we can determine the probability that their children will suffer from a particular genetic disease. At the same time, it can provide new information on the probability that the parents themselves will develop a genetic disease in the future. When investigating the genome of every individual, we can expect to find 2-3 genetic variations that can predispose them to serious genetic illness and 50-100 genetic variations of which the precise importance is unknown. In case the parents-to-be decide on preconception genetic screening (meant for the well-being of the planned child), the results can give them new information about the risk that they will become affected by a genetic disease.” The statements were categorized into eight sections (Table 2).

**Table 2:** Parental attitude toward extended pre-conceptional genomic screening

Section/question		Totally disagree	Disagree	Neither agree nor disagree	Agree	Totally agree	M†	Me†	Positive attitude (answer 4 or 5), %		
Section 1: General interest in genetic screening											
Q1	I would like to determine whether my partner or I carry any common genetic variants that could potentially lead to a genetic disease in our future child	38 (7.7)	30 (6.1)	85 (17.3)	116 (23.6)	223 (45.3)	3.9	4	68.9		
Section 2: Interest based on genetic disease/variant type											
Q2	Before becoming pregnant, I would like to ascertain whether I am a carrier of genetic diseases that are most common in Slovene population (eg, cystic fibrosis)	15 (4.1)	12 (3.3)	38 (10.3)	106 (28.7)	198 (53.7)	4.3	5	82.4		
Q3	Before becoming pregnant, I would like to ascertain whether I am a carrier of any of the thousands of genetic diseases	18 (4.9)	16 (4.3)	43 (11.7)	118 (32.1)	173 (47.0)	4.1	4	79.1		
Q4	In the context of genetic preconceptional screening, I would like to obtain information regarding genetic variants that have the potential to cause developmental abnormalities in the fetus	4 (1.1)	8 (2.2)	27 (7.5)	107 (29.6)	215 (59.6)	4.4	5	89.2		
Q5	In the context of genetic preconceptional screening, I would like to obtain information regarding genetic variants that can potentially cause diseases in my future offspring	6 (1.7)	12 (3.3)	45 (12.5)	109 (30.2)	189 (52.4)	4.3	5	82.5		
Q6	I would like to determine my carrier status for treatable or partially treatable genetic diseases (eg, breast or colon cancer)	12 (3.3)	11 (3.1)	53 (14.7)	104 (28.9)	180 (50.0)	4.2	4.5	78.9		
Q7	I would like to determine my carrier status for non-treatable diseases (eg, Huntington and Parkinson disease)	9 (2.5)	9 (2.5)	37 (10.3)	81 (22.5)	224 (62.2)	4.4	5	84.7		
Section 3: Decision in the case of a positive preconceptional screening result											
Q8	If the preconceptional genetic screening test were to confirm a pathologic genetic variant in the fetus, I would opt for prenatal diagnostics in the case of spontaneous conception	20 (5.7)	15 (4.3)	65 (18.5)	100 (28.5)	151 (43.0)	4.0	4	71.5		
Q9	If the preconceptional genetic screening test were to confirm a pathologic genetic variant in the fetus, I would opt for preimplantation genetic diagnosis	17 (4.9)	28 (8.0)	82 (23.5)	90 (25.8)	132 (37.8)	3.8	4	63.6		
Q10	If the preconceptional genetic screening test were to confirm a pathologic genetic variant in the fetus, I would choose to terminate the pregnancy	27 (7.7)	28 (8.0)	119 (33.9)	79 (22.5)	98 (27.9)	3.6	4	50.4		
Section 4: Perceived benefits of genetic screening											
Q11	I believe that preconceptional genetic screening tests are important	21 (4.6)	27 (5.9)	115 (25.1)	127 (27.7)	168 (36.7)	3.9	4	64.4		
Q12	I believe that preconceptional genetic screening tests are comforting	41 (9.1)	46 (10.2)	152 (33.8)	111 (24.7)	100 (22.2)	3.4	3	46.9		
Q13	I believe that preconceptional genetic screening tests are useful	14 (3.1)	26 (5.7)	112 (24.4)	129 (28.1)	178 (38.8)	3.9	4	66.9		
Section 5: Emotional implications											
Q14	I believe that the results of genetic testing provide us with an increased sense of safety	24 (4.9)	39 (8.0)	107 (22.0)	143 (29.4)	173 (35.6)	3.8	4	65.0		
Q15	I believe that the results of genetic testing can contribute to increased feelings of anxiety and fear	28 (5.8)	27 (5.6)	105 (21.6)	157 (32.3)	169 (34.8)	3.9	4	67.1		
Section 6: Psychosocial implications											
Q16	If I were to discover that I am a carrier of genetic disease, it would significantly impact my attitude toward life, work, and family	53 (11.1)	29 (6.1)	145 (30.3)	151 (31.5)	101 (21.1)	3.5	4	52.6		
Q17	I believe that the results of genetic testing could potentially affect the relationship I have with my partner	99 (20.6)	89 (18.5)	146 (30.4)	90 (18.7)	57 (11.9)	2.8	3	30.6		
Q18	I believe that society would treat me differently (eg, employability, insurance issues), if I were to have a child with a genetic disease	51 (10.7)	69 (14.4)	129 (27.0)	122 (25.5)	107 (22.4)	3.4	3	47.9		
Section 7: Financial aspect											
Q19	Would you be willing to pay for preconceptional carrier screening test?	51 (10.7)	57 (11.9)	159 (33.3)	128 (26.8)	82 (17.2)	3.3	3	44.0		
Q20	I would pay: 1 – 100 €; 2 – 1000 €; 3 – 5000 €; 4 – more	270 (65.7)	128 (31.1)	7 (1.7)	6 (1.6)	–	–	–	–		
Q21	The test should be covered by the national health insurance policy	12 (2.5)	25 (5.3)	74 (15.6)	84 (17.7)	279 (58.9)	4.3	5	76.6		
Section 8: Informative paragraph											
Q22	Does the information conveyed in the informative paragraph* affect respondents' decision to participate in preconceptional genetic screening?	40 (8.6)	31 (6.7)	147 (31.7)	125 (27.0)	120 (25.9)	3.6	4	52.9		

*Described in the Respondents and Methods section.

†M – arithmetic mean; Me – median.

### Statistical analysis

Descriptive statistics were employed to present the questionnaire results. The normality of data distribution was assessed visually by means of a quantile-quantile plot. The relationship between demographic factors and respondents’ attitudes was evaluated with a χ^2^ test or a Fisher exact test. To assess the differences in genetic knowledge score and responses to Likert items across demographic groups, independent-samples *t* tests and analysis of variance were conducted. Multiple pairwise comparisons were performed with the Hochberg test. A *P* value of <0.05 was considered statistically significant. The analysis was performed with SPSS 25.0 (IBM Corp., Armonk, NY, USA).

## RESULTS

### Demographics

A total of 497 respondents took part in the survey (197 couples, comprising a male and a female partner). The respondents’ demographics are outlined in Table 1.

### Genetic literacy

Individuals with higher education (college or more) achieved a higher average genetic literacy score compared with those with lower education (11.2 vs 10.3; *P* = 0.004). Furthermore, respondents with a higher mean genetic literacy score displayed more interest in general genetic screening (Q1, *P* = 0.006), screening for common genetic diseases (Q2, *P* = 0.016), and screening for variants that may cause developmental abnormalities of the fetus or diseases in the offspring (Q4, *P* = 0.008) (Table 1).

Respondents with a higher score were less supportive of pregnancy termination in the case of pathological prenatal screening results (Q10, *P* = 0.023). They also felt less secure about the test results (Q14, *P* = 0.018) and were less likely to believe that genetic screening should be covered by national health insurance policies (Q21, *P* = 0.013).

### Interest in genetic screening

The responses to all the questions in the questionnaire are shown in Table 2.

*Section 1 – General interest in genetic screening to prevent heritable diseases in offspring.* Among the 497 respondents, 339 (69%) expressed general interest in genetic screening, 153 (31%) showed no interest or had a neutral stance, while five respondents did not answer this question. The responses to this question were significantly influenced by religiousness (*P* = 0.002), level of education (*P* = 0.003), and age (*P* = 0.046). Specifically, more non-religious respondents than practicing religious respondents showed interest in genetic screening (77.6% vs 59.8%). Also, interest in genetic screening was shown by more respondents with college or higher education than those with lower education (74.2% vs 60.3%). Respondents in the age group of 36 and above showed higher interest in genetic screening than those in the age group of 18-25 (mean 4.1 vs 3.5).

The general interest in genetic screening was positively associated with perceived benefits, feelings of safety provided by the test results, and willingness to participate financially (*P* < 0.001 for each). Additionally, the respondents who believed that the genetic test should be covered by national health insurance policy also demonstrated greater interest in genetic screening (*P* = 0.032). Conversely, the extent of anxiety regarding the test results was inversely related to the general interest in genetic screening (*P* = 0.008).

Only those respondents who agreed or totally agreed to the question in Section 1 were asked to respond to the items in Sections 2 and 3.

*Section 2 – Interest in genetic screening based on a genetic disease*. A significant proportion of those who expressed general interest in genetic screening (79%-89%) demonstrated a positive attitude toward all types of genetic screening tests regardless of the specific genetic disease or variant being targeted. This included screening for common or all known diseases, treatable or untreatable diseases, and developmental abnormalities in the fetus or genetic diseases in the offspring. More non-religious respondents compared with practicing religious respondents showed interest in screening for all known genetic diseases and untreatable diseases (86.7% vs 67.9%, *P* = 0.002, and 90.9% vs 75.9%, *P* = 0.008, respectively). Respondents with college or higher education exhibited higher interest in screening for common diseases, developmental abnormalities in the fetus, disease in offspring, and untreatable diseases (87.0%, 92.2%, 87.0%, and 87.8%, respectively) compared with those with lower education (74.0%, 82.6%, 74.4%, and 78.3%, respectively; *P* values: 0.005, 0.009, 0.007, and 0.043, respectively). Furthermore, respondents aged 36 and above showed more support for screening for common diseases compared with younger individuals (mean 4.4 vs 3.8, *P* = 0.020). More of them also expressed support for screening for developmental abnormalities, diseases in the offspring, and untreatable diseases (97.1%, 85.7%, and 88.6%, respectively) compared with their younger counterparts (65.5%, 62.1%, 58.6%, respectively; *P* values: <0.001, 0.010, and <0.001, respectively).

*Section 3 – Decision in the case of a positive pre-conceptional screening result.* Those who expressed general interest in genetic screening expressed strong interest in both prenatal and preimplantation genetic screening in the event of a pathological prenatal screening result (71.5% and 63.3%, respectively). Respondents aged 26-35 years were significantly more supportive of prenatal diagnostics compared with younger respondents (74% vs 48.1%, respectively; *P* = 0.018). Additionally, respondents with higher education and non-religious respondents exhibited higher levels of support for preimplantation genetic screening compared with their counterparts (68% vs 55.2%, *P* = 0.042 for educational level; 72.8% and 51.3%, *P* = 0.004 for religiousness).

When asked about the termination of pregnancy in the case of a pathological prenatal screening result, half of the respondents answered that they would most likely or definitely terminate the pregnancy. A third of the respondents remained undecided. The responses were significantly influenced by religiousness (*P* = 0.009), with mean scores varying among religious groups: 3.1, 3.5, and 3.8 for practicing religious, non-practicing religious, and non-religious group, respectively. In percentage terms, 60% of non-religious respondents would opt for termination, compared with 40% in the religious group (*P* = 0.01).

*Section 4 – Perceived benefits of genetic screening.* Overall, 64.4% of the respondents believed that pre-conceptional screening tests were important and 66.9% found them useful. Less than half of the respondents (46.9%) considered the tests to be comforting, and a third remained undecided. Significantly more women than men believed that these tests were important (68.1% vs 59%; *P* = 0.045), useful (70.8% vs 61.1%; *P* = 0.03), and comforting (51.3% vs 40.5%; *P* = 0.024). Significantly more non-religious respondents viewed the tests as important (70.9%) and useful (73.3%) compared with those in the practicing religious group (55.9% and 59.5%, respectively; *P* values: 0.03 and 0.045, respectively).

*Section 5 – Emotional implications*. Approximately two-thirds of the respondents either agreed or strongly agreed with the statements that the results of genetic tests increased feelings of safety (65%) but also anxiety and fear (67.1%). More women than men agreed that these tests increased anxiety and fear (71.1% vs 61%; *P* = 0.020), as well as more respondents with lower education compared with those with the highest education level (73.8% vs 63.7%; *P* = 0.040).

*Section 6 – Psychosocial implications.* Overall, 31%-53% respondents believed that genetic test results could have psychological effects on their private or social life. Fewer patients from the first pregnancy group agreed or strongly agreed that genetic results could affect the relationship with their partner compared with those who already had one or more pregnancies (27.7% vs 42.1%; *P* = 0.007). Fewer respondents in the first pregnancy group agreed or strongly agreed that genetic results could affect employability and insurance issues compared with those who already had one or more pregnancies (45.5% vs 58.5%; *P* = 0.007).

*Section 7 – Financial aspect*. The majority of respondents (76.6%) believed that genetic tests should be covered by national health insurance policies. Respondents who already had one or more children were more supportive of the statement that the cost should be fully covered by national insurance company compared with respondents in their first pregnancy (86.2% vs 74.1%, respectively; *P* < 0.001). A total of 44% respondents were willing to financially participate in pre-conceptional carrier screening tests, and 65.7% were ready to pay between 1 and 100 €.

The respondents who were more supportive of the statements that genetic screening tests were important, comforting, useful, provided an improved sense of security, affected the attitude toward life, work, and family, and should be paid by users were more likely to pay 1000 € or more compared with those who disagreed or had a neutral opinion about these statements (37.4% vs 25.7%, *P* = 0.025; 40.5% vs 26.8%, *P* = 0.004; 39.7% vs 20%, *P* < 0.001; 38.2% vs 25.4%, *P* = 0.011; 43.1% vs 23.6%, *P* < 0.001; and 46% vs 23.8%, *P* < 0.001, respectively).

*Section 8 – Relevance for parent’s health*. Overall, 52.9% of participants agreed or strongly agreed that the knowledge that genetic screening results could also provide information about their own risk of developing a genetic disease would affect their decision to undergo genetic testing. One-third of the respondents were undecided on this matter. None of the demographic factors significantly influenced the responses to this statement.

## DISCUSSION

### Overall results

The study found most of the participants to be interested in genetic carrier screening. Two demographic factors directly affected this interest level. Religiosity diminished the interest in genetic carrier screening, while higher educational level increased it.

As anticipated, religious status strongly affected responses regarding termination of pregnancy if a pathogenic variant was to be prenatally identified in the fetus. However, as we asked about a hypothetical situation without providing data on the severity of the predicted disease, it is not surprising that around a third of the respondents across all religious groups were undecided. Nevertheless, 30% of practicing religious respondents denied the possibility of abortion, compared with only a minority of non-religious and non-practicing religious respondents. These statements were answered only by respondents interested in genetic screening. Since the level of interest in genetic screening was affected by religious beliefs, full participation might have yielded different results. However, we generally focused on the preferences of those with the highest interest in genetic screening tests.

As expected, interest in genetic screening was positively related to the overall opinion about genetic tests (items from section 4 – perceived benefits) and the belief in the sense of security that the test results can provide. Respondents who showed greater interest in genetic screening were also more willing to pay for the service and believed that national health insurance should fund it. Conversely, those who experienced higher levels of anxiety regarding test results and feared potential pathologic findings were less interested in genetic screening.

The majority of respondents believed that genetic test results could impact their perspectives on life, work, and family, as well as the relationship with their partner. The responses in this section showed less positive attitudes than responses in the other sections, likely due to the significant implications associated with the items. Importantly, the results in this section were not related to any of the items assessing the interest in genetic screening. This suggests that while respondents were aware of the potential psychosocial implications of genetic tests, these considerations did not have a major role in their decision to undergo genetic carrier screening tests. These results suggest that interest in genetic screening is driven mainly by the reassurance provided by a negative test result. On the other hand, it is discouraged by the fear of a confirmed pathological finding and the difficulty of making decisions regarding the termination of pregnancy.

Although there are still significant challenges and barriers to the successful implementation of expanded carrier screening programs, including the lack of understanding of genetic conditions among the general public (15), other studies have reported relatively high interest in genetic screening but also reluctance due to fear (8,16,17). Furthermore, in a study that assessed couples’ experience with expanded carrier screening, all participants stated that they would opt for the test again, and 80% would recommend it to others (18).

### Effect of genetic knowledge

Respondents’ general genetic knowledge was relatively high, which is similar to some studies (12,16), although other authors reported low genetic literacy among both patients and medical practitioners (8).

As expected, participants with higher education levels scored higher on genetic literacy. A higher genetic literacy score was inversely related to the support for pregnancy termination and the feelings of safety. Additionally, higher scores indicated a greater overall interest in genetic screening. Education influenced the general interest in genetic screening in two ways: first, through its impact on the genetic score, and second, by directly reducing fear surrounding the test results. While the former had a diminishing effect, higher education was generally associated with greater interest in genetic screening. A positive relationship between knowledge and interest in genetic screening was also reported by other studies (16,19).

Half of our respondents stated that the informative paragraph provided alongside question 22 significantly affected their decision regarding genetic screening tests, while in less than 15% the interest remained unchanged. The informative paragraph provided participants with new reasons to question the predictive value of genetic tests, such as the presence of numerous genetic variations with unknown significance. It also alerted them to potential concerns, such as the possibility of receiving information about their own risk for genetic disease. This item can be interpreted as a measure of the disparity between initial expectations and the realistic utility of existing genetic screening tests. This disparity indicates an imbalance between perceived and factual knowledge, which has also been observed in other studies (20,21). Taken together, the results concerning the informative paragraph revealed that increased knowledge about the limitations of current genetic screening tests tends to decrease interest in undergoing testing. In a realistic scenario, individuals who receive appropriate pre-test counseling may experience a reduction in their initial interest in genetic screening due to a better understanding of the subject matter and potential limitations. The association between knowledge and attitude is complex and was discussed also in other studies (12,22,23). A better-informed public does not necessarily equate to higher levels of support. In the words of Jallinoja et al (23): “Among those with the highest level of knowledge, there was both more enthusiasm and more skepticism than among those with the lowest level of knowledge.”

Our study has some limitations. The findings may not be applicable to the general population as the sample only included expectant parents who attended a prenatal education course. Attendance in this course is voluntary, so only interested parents participated. It is possible that different results regarding the genetic knowledge of parents and their attitudes toward genetic testing could be obtained compared with those in this study. However, our findings offer valuable insights into parents' attitudes toward pre-conceptional screening, especially since no similar studies have been conducted in Slovenia to date. Additionally, the current study assessed a hypothetical scenario of pre-conceptional screening. It is possible that parents might have a different view if they were faced with an actual decision.

In conclusion, the study emphasizes the significant interest in extended pre-conceptional genomic carrier screening among pregnant couples. Despite acknowledging potential challenges associated with genetic testing, the participants generally exhibited a positive attitude toward the carrier screening.

## References

[1] Grody WW Cutting GR Klinger KW Richards CS Watson MS Desnick RJ Laboratory standards and guidelines for population-based cystic fibrosis carrier screening. Genet Med 2001 3 149 54 10.1097/00125817-200103000-00010 11280952

[2] Kaback MM Population-based genetic screening for reproductive counseling: the Tay-Sachs disease model. Eur J Pediatr 2000 159 Suppl 3 S192 5 10.1007/PL00014401 11216898

[3] Cousens NE Gaff CL Metcalfe SA Delatycki MB Carrier screening for beta-thalassaemia: a review of international practice. Eur J Hum Genet 2010 18 1077 10.1038/ejhg.2010.90 20571509 PMC2987452

[4] Counsyl. Myriad Genetics. 2023. Available from: h*ttps://myriad.com/womens-health/**.* Accessed: May 11, 2024.

[5] Borry P Cornel MC Howard HC Where are you going, where have you been: a recent history of the direct-to-consumer genetic testing market. J Community Genet 2010 1 101 6 10.1007/s12687-010-0023-z 21475669 PMC3063844

[6] Patch C Sequeiros J Cornel MC Genetic horoscopes: is it all in the genes? Points for regulatory control of direct-to-consumer genetic testing. Eur J Hum Genet 2009 17 857 9 10.1038/ejhg.2008.246 19259126 PMC2986502

[7] Borry P Henneman L Lakeman P ten Kate LP Cornel MC Howard HC Pre-conceptional genetic carrier testing and the commercial offer directly-to-consumers. Hum Reprod 2011 26 972 7 10.1093/humrep/der042 21362685 PMC3079469

[8] Henneman L Borry P Chokoshvili D Cornel MC van El CG Forzano F Responsible implementation of expanded carrier screening. Eur J Hum Genet 2016 24 e1 12 10.1038/ejhg.2015.271 26980105 PMC4867464

[9] European Society of Human Genetics Statement of the ESHG on direct-to-consumer genetic testing for health-related purposes. Eur J Hum Genet 2010 18 1271 3 10.1038/ejhg.2010.129 20736974 PMC3002858

[10] Sagaser KG Malinowski J Westerfield L Proffitt J Hicks MA Toler TL Expanded carrier screening for reproductive risk assessment: An evidence-based practice guideline from the National Society of Genetic Counselors. Journal of Genetic Counseling. J Genet Couns 2023 32 540 57 10.1002/jgc4.1676 36756860

[11] Henneman L Vermeulen E van El CG Claassen L Timmermans DRM Cornel MC Public attitudes towards genetic testing revisited: comparing opinions between 2002 and 2010. Eur J Hum Genet 2013 21 793 9 10.1038/ejhg.2012.271 23249955 PMC3722681

[12] Etchegary H Public attitudes toward genetic risk testing and its role in healthcare. Per Med 2014 11 509 22 10.2217/pme.14.35 29758777

[13] Etchegary H Wilson B Rahman P Simmonds C Pullman D Public interest in whole genome sequencing and information needs: an online survey study. Per Med 2020 17 283 93 10.2217/pme-2019-0136 32589098

[14] Pereira N Wood M Luong E Briggs A Galloway M Maxwell RA Expanded genetic carrier screening in clinical practice: a current survey of patient impressions and attitudes. J Assist Reprod Genet 2019 36 709 16 10.1007/s10815-019-01414-z 30761454 PMC6505014

[15] Kraft SA Duenas D Wilfond BS Goddard KAB The evolving landscape of expanded carrier screening: challenges and opportunities. Genet Med 2019 21 790 7 10.1038/s41436-018-0273-4 30245516 PMC6752283

[16] LePoire E Basu B Walker L Bowen DJ What do people think about genetics? A systematic review. J Community Genet 2019 10 171 87 10.1007/s12687-018-0394-0 30406598 PMC6435762

[17] Etchegary H Public attitudes toward genetic risk testing and its role in healthcare. Per Med 2014 11 509 22 10.2217/pme.14.35 29758777

[18] van Dijke I Lakeman P Sabiri N Rusticus H Ottenheim CPE Mathijssen IB Couples’ experiences with expanded carrier screening: evaluation of a university hospital screening offer. Eur J Hum Genet 2021 29 1252 8 10.1038/s41431-021-00923-9 34155360 PMC8384865

[19] Henneman L Bramsen I Van der Ploeg HM Adèr HJ Van der Horst HE Gille JJP Participation in preconceptional carrier couple screening: Characteristics, attitudes, and knowledge of both partners. J Med Genet 2001 38 695 703 10.1136/jmg.38.10.695 11594339 PMC1734747

[20] Khdair SI Al-Qerem W Jarrar W Knowledge and attitudes regarding genetic testing among Jordanians: An approach towards genomic medicine. Saudi J Biol Sci 2021 28 3989 99 10.1016/j.sjbs.2021.04.004 34220256 PMC8241592

[21] Oliveri S Masiero M Arnaboldi P Cutica I Fioretti C Pravettoni G Health Orientation, knowledge, and attitudes toward genetic testing and personalized genomic services: preliminary data from an Italian sample. BioMed Res Int 2016 2016 e6824581 10.1155/2016/6824581 28105428 PMC5220460

[22] Etchegary H Pullman D Simmonds C Rabie Z Rahman P Identifying aspects of public attitudes toward whole genome sequencing to inform the integration of genomics into care. Public Health Genomics 2021 24 229 40 10.1159/000515952 34038902

[23] Jallinoja P Aro AR Does knowledge make a difference? The association between knowledge about genes and attitudes toward gene tests. J Health Commun 2000 5 29 39 10.1080/10810730050019546 10848030

